# Land Resource Use Classification Using Deep Learning in Ecological Remote Sensing Images

**DOI:** 10.1155/2022/7179477

**Published:** 2022-04-21

**Authors:** Bin Xia, Fanyu Kong, Jun Zhou, Xin Wu, Qiong Xie

**Affiliations:** ^1^Department of Management, Chengyi University College, Jimei University, Xiamen, Fujian 361021, China; ^2^Chongqing Engineering Technology Research Center for Development Information Management, Chongqing Technology and Business University, Chongqing, 400067, China; ^3^Chongqing Business Vocational College, Chongqing, 401331, China

## Abstract

Aiming at the problems that the traditional remote sensing image classification methods cannot effectively integrate a variety of deep learning features and poor classification performance, a land resource use classification method based on a convolutional neural network (CNN) in ecological remote sensing images is proposed. In this study, a seven-layer convolution neural network is constructed, and then the two fully connected layer features of the improved CNN network training output are fused with the fifth layer pooled layer features after dimensionality reduction by principal component analysis (PCA), so as to obtain an effective remote sensing image feature of land resources based on deep learning. Further, the classification of land resources remote sensing images is completed based on a support vector machine classifier. The remote sensing images of Pingshuo mining area in Shanxi Province are used to analyze the proposed method. The results show that the edge of the recognized image is clear, the classification accuracy, misclassification rate, and kappa coefficient are 0.9472, 0.0528, and 0.9435, respectively, and the model has excellent overall performance and good classification effect.

## 1. Introduction

The remote sensing image is a comprehensive image reflecting various surface information obtained by sensors. The research on target classification of large-area remote sensing images is not only an important way to obtain land cover information but also provides important basic support for its application in the fields of sea situation monitoring, urban planning, environmental supervision, rescue, disaster relief, and military reconnaissance; it is of great significance both from the perspective of social economy and ecological environment [1]. With the continuous development of remote sensing technology, remote sensing images now show the characteristics of hyperspectral, high space, and high resolution. The information obtained from the images is more and more comprehensive, and its application field is also expanding [2, 3]. The remote sensing image of land resources has a large amount of data, complex information, and fast update. Therefore, how to accurately extract useful land information from massive remote sensing image data by computer to achieve efficient land use is a key problem to be solved [4].

For remote sensing image target classification, the computer automatically distinguishes the attributes of pixels in remote sensing images with patterns representing certain features through a pattern recognition system, so as to obtain the classification information of remote sensing images [5]. In the research of land resource use classification using remote sensing images, researchers mostly used visual interpretation and traditional pattern recognition classification methods at first. Visual interpretation is simple, but it takes a long time, and there are personal differences, resulting in inaccurate classification [6]. Traditional classification methods include a minimum distance method, maximum likelihood method, etc. [7, 8]. Reference [9] studies feature extraction based on high-resolution remote sensing images for coastal land use planning. Through the research and analysis of space motion remote sensing image sequence, the characteristic parameters of land environment and moving objects are obtained, but the consideration of ecological factors is relatively single, which does not have good popularization. Reference [10] compares the classification results of remote sensing images in specific areas by four methods: random forest, support vector machine, regression tree, and minimum distance. Reference [11] proposed a method of normalized differential vegetation index (NDVI) using time series. Using a time series NDVI database to modify the classification results can significantly improve the classification accuracy of land cover products, but this method increases the amount of calculation and cost. Reference [12] used the object-oriented classification method combined with fuzzy classification and cart (classification and expression tree) decision tree classification method to classify the land information of Dongjiang River Basin and obtained a more accurate classification effect than the maximum likelihood method and unsupervised classification method. Although the pattern recognition classification method overcomes some shortcomings of visual interpretation, it is not good at extracting spatial information and has poor flexibility.

With the development of remote sensing technology and computer technology, many new classification methods are gradually emerging, mainly including artificial neural network (ANN), support vector machine (SVM), and fuzzy theory and expert system [13, 14]. Reference [15] proposed a remote sensing image classification method combining SVM and k-nearest neighbor. Using the class separability of SVM and the spatial and spectral characteristics of remote sensing data, a distance formula is designed as the measurement standard considering vector brightness and direction, which effectively realizes the accurate classification of remote sensing images, but the classification efficiency is low. Reference [16] uses the land segmentation method of remote sensing image based on the convolutional neural network to realize the correct marking of different land cover types. However, for remote sensing images with complex background, more and larger database learning and training are needed to better complete the classification task. Aiming at the problem that traditional remote sensing image classification methods are vulnerable to the loss of spatial features, reference [17] proposed an image semantic segmentation method based on a dense coordinate transformation network, which improves the accuracy of semantic segmentation of high-resolution remote sensing images but still has a certain dependence on the training data set. Reference [18] proposed a feature integration network including multiscale features and enhancement stages for the classification of land remote sensing images and used two-dimensional extended convolution with different sampling rates for each scale feature layer to realize image classification with higher accuracy than ordinary depth learning methods, but the classification efficiency needs to be further improved.

The improvement of most classification algorithms can improve the accuracy of land resources classification, but still, there are problems such as too large processing scale, complex calculation, and easy to fall into the minimum. In particular, it is difficult to meet the needs of current applications in classification efficiency and speed and cannot well solve many problems of high spectral remote sensing images for land resources [19]. Therefore, this study proposes a land resource use classification method using deep learning in ecological remote sensing images. The innovations of this study are summarized as follows:

1. In this study, three high-level features of remote sensing images are extracted by using the convolutional neural network (CNN), and a variety of depth image features are fused in series. The fused features cover more complete information and have stronger discrimination.
2. To further improve the classification performance, the proposed method designs a remote sensing image classifier based on SVM, which combines deep learning features with a deep classifier to solve the problem of poor classifier performance.

## 2. Study Area and Data

The visible shortwave infrared hyperspectral camera carried by the “Gofen 5” (GF-5) satellite has a spectral resolution of 5–10 nm, a spatial resolution of 30 m, and a width of 60 km. The camera can simultaneously obtain the spatial information and spectral information of 330 continuous spectral segments of ground objects in the range of 400–2500 nm. The collected data are mainly composed of two parts: visible near infrared (VNIR) and short wave infrared (SW). Among them, VNIR has 150 bands and SW has 180 bands, a total of 330. The VNIR band range is about 0.39–1.03, the spectral resolution is 5 nm, the SW band range is about 1.0–2.5, and the spectral resolution is about 10 *μ*m.

**Figure 1:**
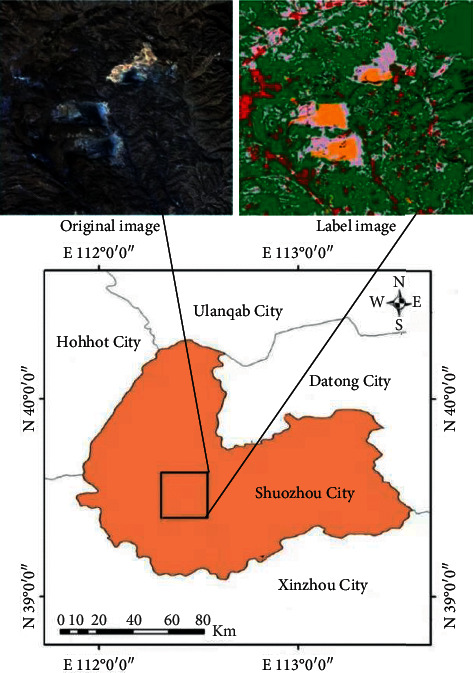
Geographical location of the study area.

The study area of this study is located in the Pingshuo mining area, Shanxi Province, covering about 400 km^2^, N39°24′52″–39°37′15″, and E 112°16′29″–112°33′43″. This area is the largest open-pit coal mine in China, and the ecological environment has been damaged due to perennial mining. Therefore, it is of great significance to study the land cover types in this area. The data used in the proposed method are visible short wave infrared hyperspectral data of GF-5 satellite, with a total of 4 images. The corresponding high-resolution image of “Gaofen-2” satellite with the closest region (the spatial resolution of the fused image is 0.8 m) and the global 30 m land cover type map were obtained free of charge from Tsinghua University. First, atmospheric correction is carried out to remove the impact of atmosphere on the image. Then, referring to the thematic map of land cover types with 30 m spatial resolution, the land cover types are manually drawn on high-resolution images using the Environment for Visualizing Images (ENVI) platform. Finally, the coverage type map is downsampled to 30 m resolution as the real label of land cover in this area, as shown in Figure 1.

## 3. Research Method

### 3.1. System Model

**Figure 2:**
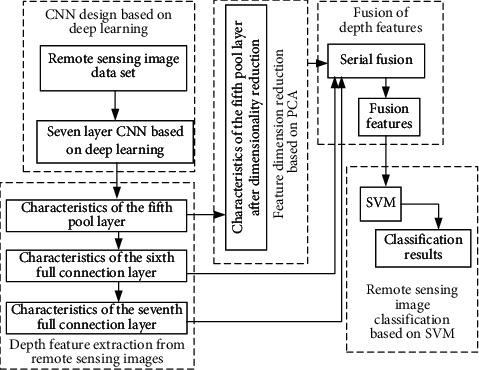
The overall architecture of the proposed method.

The proposed method first designs a seven-layer CNN and then inputs high-resolution remote sensing image samples into the network for training. The specific steps are as follows:

1. Build multiclass remote sensing image sample data set *I*=[*I*_1_, *I*_2_,…, *I*_*i*_,…, *I*_*N*_] and make the corresponding sample label *L*=[*L*_1_, *L*_2_,…, *L*_*i*_,…, *L*_*N*_], where *N* represents a class *N* remote sensing image, *I*_*i*_(*i*=1,2,…, *N*) represents a collection of class *i* remote sensing images, and *L*_*i*_ represents a label collection of class *i* remote sensing images.
2. The remote sensing image data set is divided into training set and test set. For each type of remote sensing image in the data set, the total number of samples is *n*, from which *m* images are randomly selected to build a training set, and the other *n* − *m* images to build a test set, as follows: *Tr*=[*Tr*_1_, *Tr*_2_,…, *Tr*_*i*_,…, *Tr*_*N*_] and *Te*=[*Te*_1_, *Te*_2_,…, *Te*_*i*_,…*Te*_*N*_], where *Tr*_*i*_ represents the training set of class *i* remote sensing images, including *m* images; *Te*_*i*_ represents the test set of class *i* remote sensing images, including *n* − *m* images.
3. Build a seven-layer CNN model. The first five layers are represented by the first layer, the second layer, the third layer, the fourth layer, and the fifth layer, respectively. The first layer, the second layer, and the fifth layer contain convolution layer and pool layer. Each convolution layer is represented by Conv1, Conv2, and Conv5, respectively, and each pool layer is represented by Pool1, Pool2, and Pool5, respectively. Both the third and fourth layers have only one convolution layer, which is represented by Conv3 and Conv4. The sixth and seventh layers are all connected layers, represented by FC6 and FC7, respectively. The overall architecture of remote sensing land image classification method based on a 7-layer CNN network structure is shown in Figure 2.
4. The training samples of remote sensing images are used to train the CNN. First, the remote sensing image training set is input into the built CNN to calculate the output value of each neuron of CNN.

Assuming that layer *l* is a convolution layer, the calculation of the *j* feature map *y*_*j*_^*l*^ of layer *l* is as follows:

(1) $$\begin{array}{c} y_{j}^{l}=\delta \left(\sum_{i\in M^{l-1}}y_{i}^{l-1}*\kappa_{ij}^{l}+b_{j}^{l}\right), \end{array}$$

where *∗* is convolution operation, *y*_*i*_^*l*−1^ is the *i* feature map of layer *l* − 1, *κ*_*ij*_^*l*^ is the convolution kernel used for connection between *y*_*i*_^*l*−1^ and *y*_*j*_^*l*^, *b*_*j*_^*l*^ is the offset of *y*_*j*_^*l*^, *δ*() is the activation function, and *M*^*l*−1^ is the number of feature maps of layer *l* − 1.

Assuming that layer *l* is a pool layer, the calculation of the *j* feature map *y*_*j*_^*l*^ of layer *l* is as follows:

(2) $$\begin{array}{c} y_{j}^{l}=\delta \left(\alpha_{j}^{l}\cdot f\left(y_{j}^{l-1}\right)+b_{j}^{l}\right), \end{array}$$

where *α*_*j*_^*l*^ is the pooling parameter of *y*_*j*_^*l*^, *y*_*i*_^*l*−1^ is the *j* feature map of layer *l* − 1, *f*() is the pooling function, and *b*_*j*_^*l*^ is the offset of *y*_*j*_^*l*^.

Assuming that layer *l* is a fully connected layer, the calculation of the *j* feature map *y*_*j*_^*l*^ of layer *l* is as follows:

(3) $$\begin{array}{c} y_{j}^{l}=\delta \left(y^{l-1}+b_{j}^{l}\right), \end{array}$$

where *y*^*l*−1^ is the weighted result of all feature maps of layer *l* − 1 and *b*_*j*_^*l*^ is the offset of *y*_*j*_^*l*^.

Second, the overall loss function of CNN is calculated. Any one of the label samples *G*_*i*_(*i*=1,2,…, *N* × *m*) is set in the remote sensing image training set. The label of *G*_*i*_ is actually a one-of-*N* label. That is, for sample *G*_*i*_, its classification label is as follows:

(4) $$\begin{array}{c} \vartheta_{i}^{k}=\left\{\begin{array}{cc} 1, & G_{i}\text{ actually belongs to category }k,\\ 0, & G_{i}\text{ is not actually a class }k. \end{array}\right. \end{array}$$

For label sample *G*_*i*_, if the probability of model identification of class *k*(*k*=1,2,…, *N*) is *p*_*i*_^*k*^, then the error is defined as *E*_*i*_:

(5) $$\begin{array}{c} E_{i}=\frac{1}{2}\sum_{k=1}^{N}{\left(p_{i}^{k}-\vartheta_{i}^{k}\right)}^{2}. \end{array}$$

Based on the errors of all training samples, the loss function *φ*_*E*_ of the model is calculated as follows:

(6) $$\begin{array}{c} \varphi_{E}=\sum_{i=1}^{N\times m}E_{i}=\frac{1}{2}\sum_{i=1}^{N\times m}\sum_{k=1}^{N}{\left(p_{i}^{k}-\vartheta_{i}^{k}\right)}^{2}. \end{array}$$

Finally, the gradient descent algorithm is used to minimize the loss function and update the parameters in the network. The purpose of training CNN is to find the optimal parameters to minimize the loss function *φ*_*E*_. The parameters of CNN are *κ*_*ij*_^*l*^, *α*_*j*_^*l*^, and *b*_*j*_^*l*^. *ψ* represents the above three parameters, that is, *ψ*=(*κ*_*ij*_^*l*^, *α*_*j*_^*l*^, *b*_*j*_^*l*^); after the CNN is trained through the remote sensing image training set, a set of parameters *ψ*^*∗*^ can be obtained as follows:

(7) $$\begin{array}{c} \psi^{*}=\arg\underset{\psi }{\min}\varphi_{E}. \end{array}$$

The gradient descent algorithm is used to update the parameter *ψ* of CNN and minimize the loss function *φ*_*E*_:

(8) $$\begin{array}{c} \psi^{\left(i\right)}=\psi^{\left(i-1\right)}-\varepsilon {\frac{\partial \varphi_{E}}{\partial \psi }|}_{\psi =\psi \left(i\right)}, \end{array}$$

where *ε* is the learning rate of CNN, which determines the adjustment range of each step; *ψ*^(*i*)^ is the updated parameter of group *i*; *ψ*^(*i* − 1)^ is the parameter of group *i* − 1; ∂*φ*_*E*_/∂*ψ* is the partial derivative of parameter *ψ* for loss function *φ*_*E*_.

### 3.2. Improved Activation Function TReLU

In this study, a TReLU activation function combining the advantages of tanh function and parametric ReLU (PReLU) function with parameters is proposed. The TReLU activation function not only retains the advantages of fast convergence speed of PReLU function and can alleviate the disappearance of gradient but also uses tanh function to introduce negative half axis activation value and its soft saturation characteristics to prevent “neuron death” and offset and is more robust to noise [20, 21].

The mathematical expression of TReLU activation function is as follows:

(9) $$\begin{array}{c} g\left(x\right)=\left\{\begin{array}{cc} x, & x>0,\\ \tanh\left(\beta x\right), & x\leq 0, \end{array}\right. \end{array}$$

where *β* is a variable parameter used to control the unsaturated region of the function.

**Figure 3:**
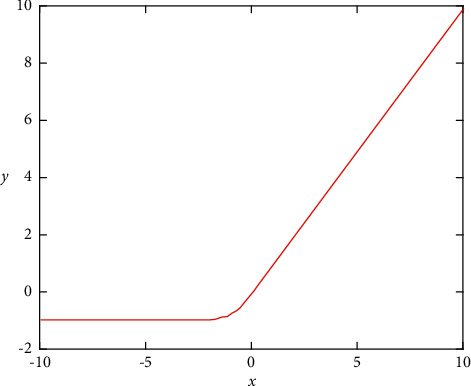
TReLU function.

The function image corresponding to TReLU is shown in Figure 3 (assuming that *β* is 1).

The initial value of *β* is set to 1. As can be seen from Figure 3, the function is approximately linear at the origin and has a fast convergence speed [22, 23]. Compared with the existing activation functions Sigmoid, ReLU, and PReLU, the proposed improved activation function has the following advantages:

1. *he Problem of Gradient Disappearance*. When *x* > 0, the derivative value of the function is always 1, so the TReLU function maintains the gradient without attenuation at *x* > 0, which alleviates the problem.
2. *Activation of Negative Values*. The TReLU function retains some gradient values in the negative half-axis unsaturated region. When the activation value falls into the unsaturated region, it can still obtain effective activation and retain the characteristics of the image. At the same time, the size of the unsaturated region is controlled by parameter *β* to activate the negative value feature more effectively [24]. In the actual training, with the continuous training, by automatically adjusting the parameters of *β*, more eigenvalues falling on the negative axis can be activated and more information can be transmitted to the front layer, alleviating the phenomenon of gradient disappearance [25].
3. *Approximation to 0-Means Distribution*. The TReLU function has an active value on the negative half axis, which ensures that the mean value of the output is approximately 0. The average value of the output of the upper layer is approximately 0, which effectively alleviates the offset of the ReLU activation function, and the weight can be updated quickly, so as to obtain a faster gradient descent speed.
4. *Robustness to Noise*. The TReLU function has soft saturation on the negative half axis when the output range of the function is [0, 1). Soft saturation means that the function can reduce the change of information output to the next layer, which is robust to noise and reduces complexity.

### 3.3. High-Level Feature Extraction

The designed CNN is used to extract multiple depth features of remote sensing images. First, the whole data set, including all sample images in the training set and test set, is input into the trained CNN, and the first five layers of features of all sample images are automatically learned through the CNN model. Among them, the convolution kernel of the first layer mainly extracts the bottom features of the image, such as edges, angles, and curves. The input of the second layer is actually the output of the first layer. The filter of this layer can be used to detect the combination of bottom features, such as semicircle and quadrilateral and these information correspond to the color, edge, contour, and other features of the image. The third layer is the image texture feature. The fourth layer of learning obtains more distinctive features, which reflect the differences between classes. The fifth layer of learning obtains complete and discriminative key features, which are a class of objects with significant differences in remote sensing images. Finally, the output result of CNN layer 5 pooling layer can be obtained, which includes all the characteristic diagrams calculated by layer 5 pooling layer [26].

Then, using equation (3), the output results *F*_6 and *F*_7 of CNN layer 6 and 7 full connection layers FC 6 and FC 7 can be obtained, including all the characteristic diagrams calculated by FC 6 and FC 7 layers. Finally, *F*_6 and *F*_7 are two different high-level features of remote sensing images.

### 3.4. Feature Dimensionality Reduction and Classification

For the output of the fifth pool layer of CNN, the principal component analysis (PCA) method is used to reduce the dimension, and the reduced dimension result is used as the third high-level feature of remote sensing image. PCA dimensionality reduction process is as follows:

1. *Matrix Deformation*. The output result of the fifth pool layer of CNN is transformed into a two-dimensional matrix *C*, and each row of the two-dimensional matrix reflects the feature vector corresponding to a remote sensing training sample.
2. *Zero Mean*. Each column of two-dimensional matrix *C* is zeroed to obtain a new matrix *C*_0_, and the average value of each column of *C*_0_ is 0.
3. *Covariance MatrixM*_0_*ofC*_0_. Covariance matrix *M*_0_ reflects the relationship between the feature vectors of all remote sensing image training samples: if the covariance of the two sample feature vectors is not 0, it indicates that there is correlation between them; otherwise, it means irrelevant. The final calculated covariance matrix *M*_0_ is a diagonal matrix with size *d* × *d*.
4. Find *d* eigenvalues and *d* eigenvectors of covariance matrix *M*_0_.
5. Select the principal component of covariance matrix *M*_0_. The eigenvectors of the covariance matrix *M*_0_ are arranged from large to small according to the eigenvalues, and the first *q* eigenvectors are selected according to the accuracy to form the principal component matrix *M*_*q*_.
6. The dimension of the output of the fifth pool layer is reduced, and the feature after dimension reduction is *F*_5=*C* · *M*_*p*_. *F*_5 is the third high-level feature of remote sensing image.

After using PCA to reduce the dimension of deep features, the enhanced deep learning features are used to train an SVM model. A multiclass SVM is defined as follows:

(10) $$\begin{array}{c} \underset{\omega ,b,s}{\min}\frac{1}{2}{\left\|\omega^{\prime }\right\|}^{2}+\tau \sum_{a=1}^{N}s_{a},\\ \text{s.t. }\delta \left(\omega^{\prime }\kappa \left(z_{a}\right)+b\right)\geq 1-s_{a},\;s_{a}\geq 0,a=1,2,\ldots ,N, \end{array}$$

where *ω*′ is the projection of multiclass SVM model; *τ* is the penalty parameter, set it to 0.01; *s*_*a*_ is a nonnegative relaxation variable; *z*_*a*_ is the enhanced feature after PCA dimensionality reduction. The depth feature of CNN is further enhanced, then the feature will be used to continue the training of the SVM classifier, and the trained SVM classifier is tested with the test set.

## 4. Experiment and Analysis

### 4.1. Experimental Environment

This experiment is based on a Tensorflow framework. Tensorflow is a powerful visualization suite of low-level and high-level interfaces (Tensorboard) for huge and active community network training. It can track network topology and performance, making debugging easier and more convenient. The specific experimental environment is listed in Table 1. On the Ubuntu 16.04 operating system, some dependent libraries are first installed, such as Python and open CV, and then the python environment and Tensorflow are installed. In addition, the graphics processing unit (GPU) mode is used. After the environment is configured, the network is built according to the designed network structure, including network structure, convolution kernel size, step length, and the number of feature maps of each layer.

### 4.2. Evaluating Indicator

The evaluation indexes include classification accuracy Acc, misclassification error, and kappa coefficient. The classification accuracy and misclassification rate are calculated as follows:

(11) $$\begin{array}{c} \text{Acc}=\frac{\text{TP}}{\text{Num}},\\ \text{Error}=1-\text{Acc}=\frac{\text{FP}}{\text{Num}}, \end{array}$$

where TP is the number of correctly classified images in the remote sensing image test set, Num represents the total number of images, and FP is the number of incorrectly classified images.

Assuming that the actual number of samples is *γ*_1_, *γ*_2_,…, *γ*_*N*_ and the predicted number is *η*_1_, *η*_2_,…, *η*_*N*_, the kappa coefficient is defined as follows:

(12) $$\begin{array}{c} R_{\text{Kappa}}=\frac{\left(\text{Acc}-P_{e}\right)}{\left(1-P_{e}\right)},\\ P_{e}=\frac{\gamma_{1}\eta_{1}+\gamma_{2}\eta_{2}+\cdots +\gamma_{N}\eta_{N}}{\text{Num}\times \text{Num}}, \end{array}$$

where Acc is the actual accuracy and *P*_*e*_ is the theoretical accuracy. The higher the kappa coefficient, the better the overall classification accuracy of the method.

### 4.3. Model Training

**Figure 4:**
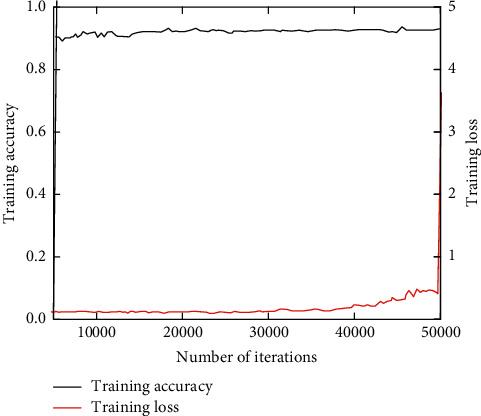
Model training accuracy and loss function.

The training set and verification set after PCA dimensionality reduction are used to train the improved CNN model. With the increase of the number of iterations, the variation trend of the training accuracy of the model and the loss function value of the training objective function are shown in Figure 4.

As can be seen from Figure 4, with the increase of iterations, the training accuracy of CNN model gradually tends to be stable, 93% of the training accuracy can be obtained at the highest, and the training loss gradually decreases and tends to be flat, indicating that the model has good convergence.

### 4.4. Land Classification Result Map

**Figure 5:**
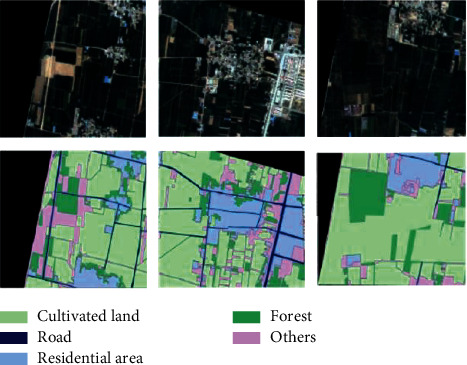
Land classification results.

On the test set samples, the proposed method is used to extract five types of land resources. The proposed method extracts three high-level features from remote sensing images and fuses them to generate the final classification map. The results are shown in Figure 5.

As can be seen from Figure 5, the five land resource use types are clearly identified, especially residential areas, which are relatively scattered and irregular, but the location of residential areas can be clearly seen in the identification result figure. The distribution of roads and cultivated land is very regular, and the overall recognition effect is ideal.

### 4.5. Comparison with Other Methods

#### 4.5.1. Comparison of Cultivated Land Classification Results

**Figure 6:**
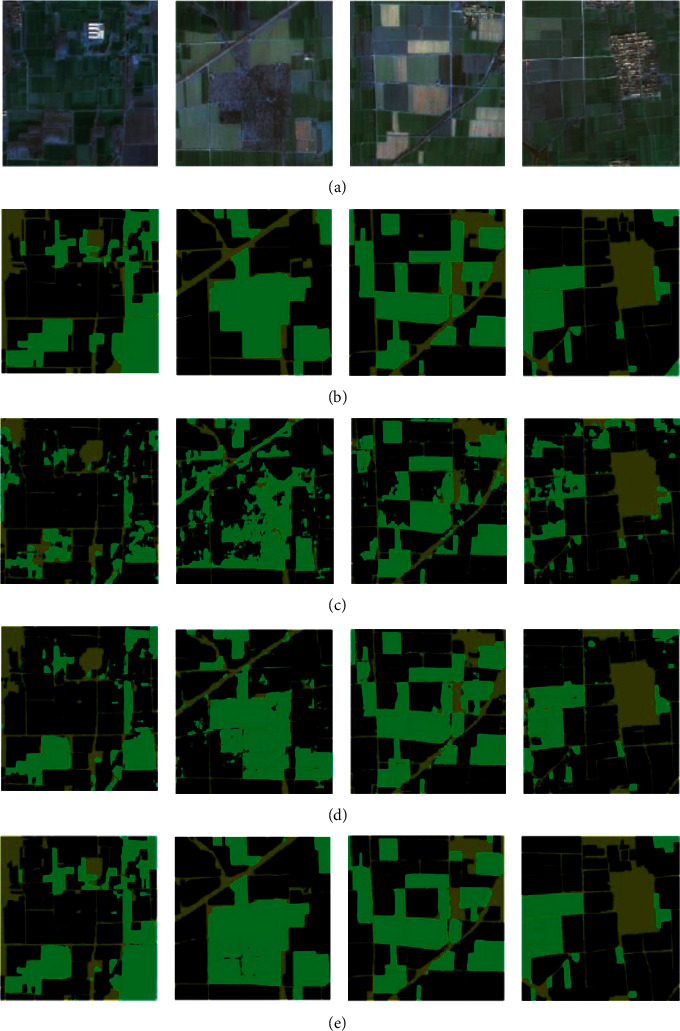
Comparison of cultivated land classification results. (a) Original image. (b) Reference [12]. (c) Reference [15]. (d) Reference [17]. (e) Proposed model.

In order to more intuitively evaluate the performance of the proposed method in cultivated land recognition, it is compared with the recognition results obtained by the methods used in references [12, 15, 17]. In the experiment, the trained model is applied to the Gaofen-5 for recognition, and the results are shown in Figure 6.

As can be seen from Figure 6(a), cultivated land has regular graphics and clear edges in the image and accounts for a very large proportion of the whole image. Reference [12] adopts the traditional fuzzy and decision tree classification, which can identify a large area of land types, but the identification effect of small land resources is poor, and the misclassification phenomenon is obvious. Reference [15] combines SVM and k-nearest neighbor to complete cultivated land recognition. Because it is not suitable for processing complex remote sensing images, there are many missing points at the edge, and there are many missing points in light-colored cultivated land. In reference [17], the depth CNN model is used to identify the cultivated land image. The most important cultivated land position is extracted accurately, but there is a case of wrong points and missing points. The proposed method can better identify the cultivated land image, and the contour is clear, which is better than other comparison methods.

#### 4.5.2. Comparison of Evaluation Indicator

The performance of the four classification methods is quantitatively analyzed. The classification accuracy Acc, misclassification error, and kappa coefficient are listed in Table 2.

It can be seen from Table 2 that the classification accuracy, misclassification rate, and kappa coefficient of the proposed method are 0.9472, 0.0528, and 0.9435, respectively, which are better than other comparison methods. The proposed method adopts the 7-layer CNN network structure, improves the activation function, reduces the dimension by PCA, and improves the classification accuracy. Reference [17] proposed a dense coordinate transformation network for image recognition based on the depth CNN model, but it has not been optimized in terms of dimensionality reduction and activation function. Compared with the proposed method, the recognition accuracy is reduced by 0.0186. Reference [15] combines SVM and k-nearest neighbor for remote sensing image classification. For complex land resource use types, the classification performance is poor, and the kappa coefficient is only 0.8839. The method used in Reference [12] is more traditional, so the classification effect is not ideal.

#### 4.5.3. Comparison of Training and Testing Time

**Figure 7:**
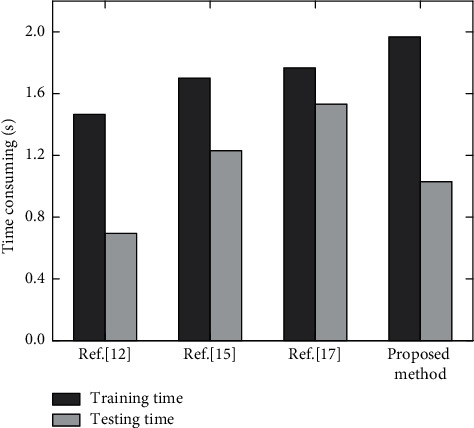
Training and testing time of different methods.

Classification efficiency is another important indicator of land resource use classification. The time consumption of the four methods on the training set and test set is shown in Figure 7.

As can be seen from Figure 7, the proposed method takes the longest time in the training phase, which is 1.95 s. This is because the method used in Reference [12] is relatively simple. The training stage of reference [15] includes only the training of k-nearest neighbor model, whereas the training stage of reference [17] includes the training of convolutional neural network and the process of feature extraction. The training stage of the proposed method includes not only the training of convolutional neural network and the extraction of three depth features but also the fusion of three depth features. In addition, in the test stage, due to the simple calculation in reference [12], the test time is only 0.72 s. The methods used in reference [14, 17] are complex, and the test time is more than 1.2 s. After training, the proposed method has the best performance in the test, and after PCA dimensionality reduction, the calculation speed is faster, and the test time is about 0.95 s. Overall, the proposed method has the best overall performance and has certain practicability in the application of land resource use classification.

## 5. Conclusion

Using a deep learning model to segment and extract ecological remote sensing images can obtain high-precision land use classification information, which plays an important role in the rational development of land resources and the development of precision agriculture. Therefore, a land resource use classification method based on deep learning in ecological remote sensing images is proposed. The remote sensing image samples are input into the seven-layer CNN model. The activation function of the model adopts the TReLU function, and the three high-level image features are fused in series and then input into the SVM classifier to complete the classification of land resources remote sensing images. The remote sensing images of Pingshuo mining area in Shanxi Province are used to analyze the proposed method. The results show that the improved CNN model can achieve rapid convergence, and the image edges recognized by the proposed method are clear. The Acc, error, and kappa coefficients are 0.9472, 0.0528, and 0.9435, respectively, and the training and testing times are 1.8 s and 0.95 s, respectively. The overall performance is better than other comparison methods.

Remote sensing images often contain complex geometric and semantic information. The next research work needs to consider not only the semantic information contained in the image itself but also some more complex factors such as occlusion, blur, and distortion. In addition, in terms of data amplification, the subsequent work can consider using the GAN model to generate some data with the same distribution as the real remote sensing image, so as to meet the requirements of the deep learning model for a large amount of training data.

## Figures and Tables

**Table 1:** System experimental environment parameters.

Environment	Parameter setting
Operating system	Ubuntu16.04
GPU	GTX TITAN X (12G)
CPU	Intel E5-2600 v3
Deep learning framework	Tensorflow
Memory	32G
Computer language	Python 3.6

**Table 2:** Evaluation indicator values of four methods.

	Reference [12]	Reference [15]	Reference [17]	Proposed method
Acc	0.8655	0.8907	0.9286	0.9472
Error	0.1345	0.1093	0.0714	0.0528
*R*_Kappa_	0.8629	0.8839	0.9193	0.9435

## Data Availability

The data included in this paper are available without any restriction.
